# Probabilistic Learning of Cue-Outcome Associations is not Influenced by Autistic Traits

**DOI:** 10.1007/s10803-022-05690-0

**Published:** 2022-08-11

**Authors:** Jia Hoong Ong, Fang Liu

**Affiliations:** School of Psychology and Clinical Language Sciences, University of Reading, Reading, UK

**Keywords:** Probabilistic, Statistical learning, Prediction, Bayesian, Predictive coding, Autistic traits

## Abstract

According to Bayesian/predictive coding models of autism, autistic individuals may have difficulties learning probabilistic cue-outcome associations, but empirical evidence has been mixed. The target cues used in previous studies were often straightforward and might not reflect real-life learning of such associations which requires learners to infer which cue(s) among many to track. Across two experiments, we compared adult learners with varying levels of autistic traits on their ability to infer the correct cue to learn probabilistic cue-outcome associations when explicitly instructed to do so or when exposed implicitly. We found no evidence for the effect of autistic traits on probabilistic learning accuracy, contrary to the predictions of Bayesian/predictive coding models. Implications for the current Bayesian/predictive coding models are discussed.

Suppose one is trying to predict if it will rain shortly without the benefit of a weather forecasting app. One might look at the clouds to see if they are grey to help make the prediction and act appropriately (e.g., grey clouds may signal rain, so one might bring an umbrella). This example, though trivial, demonstrates the necessary inferences and predictions that we make to adapt and survive in the environment. Such predictions can be understood from a Bayesian perspective (Knill & Pouget, 2004), which, broadly speaking, incorporates both the bottom-up likelihood of the input (“How likely do clouds appear grey when it rains?”) and top-down priors or expectations (“How likely does it rain generally?”) to determine the most optimal or rational prediction (i.e., the posterior probability; “How likely does it rain when there are grey clouds?”). According to predictive coding theories (Friston, 2005), when there is a discrepancy between the prediction and the outcome (e.g., seeing grey clouds and thinking it is going to rain but it does not), prediction errors are generated, which can then be used to adjust the internal generative model (e.g., lower the predictive value of grey clouds and rain) so that future predictions will be more optimal. But not all prediction errors are useful; some may simply be noise or random errors (e.g., that incident of grey clouds was not predictive of rain because of haze and smog in the atmosphere that day), and as such should be ignored and no adjustment to the internal predictive model should be made without further evidence. The ideal learner, thus, needs to accurately weigh the prediction errors on their importance to optimise their future predictions.

In recent times, various Bayesian and predictive coding models, though differing in the specifics, have been proposed to understand autism and explain myriad autistic experience such as hypo/hyper-sensitivity to sensory stimuli, intolerance to uncertainty, and restricted and repetitive behaviours (Haker et al., 2016; Palmer et al., 2017). Some suggest that autistic individuals make less use of top-down priors, and as a result, experience the world too veridically (Pellicano & Burr, 2012). Others have reached the same conclusion, but argue that, instead of attenuated priors, autistic individuals place more weight on bottom-up processes such as sensory experience and input likelihood when making inferences (Brock, 2012). As a consequence of attenuated priors and/or inappropriate weighting on bottom-up processes, one may experience a flooding of sensory information, a commonly reported autistic experience (Tavassoli et al., 2014), as each sensory perception is experienced as new or more intensely. Some propose that autistic individuals may have atypical precision of the prediction error (Cruys et al., 2014; Lawson et al., 2014) such that, they may treat all prediction errors—even those that should be ignored—as useful, leading to less optimal future inferences. Yet others propose that autistic individuals have difficulties learning regularities from the environment, which are needed to make accurate inferences (Sinha et al., 2014). Consequently, autistic individuals are said to find unpredictable situations to be challenging and thus may engage in predictable, repetitive behaviours.

These theoretical models have found some support from empirical studies that examined low-level sensory perception among autistic individuals. For example, when presented with two pairs of tones and required to judge whether the tones within the second pair had the same frequency or not, neurotypical individuals showed a bias towards the preceding tone. Specifically, their representation of the first tone of the second pair was biased by the second tone of the first pair (a so-called “contraction bias”), whereas autistic individuals were less likely to show such a bias, suggesting a more veridical perception of the tones (Jaffe-Dax & Eigsti, 2020). Electrophysiological measures have also found that autistic individuals showed less habituation in event-related responses (ERP) to repeated stimuli (Jamal et al., 2020) and less sensitivity to deviants of differing presentation frequencies as measured using mismatch negativity (MMN) in an oddball paradigm (Goris et al., 2018), which implies atypical prediction of incoming stimuli relative to neurotypical individuals. However, conflicting findings have also been reported (Finnemann et al., 2021; Knight et al., 2020; Van de Cruys et al., 2018). For instance, compared to neurotypical individuals, autistic individuals showed similar improvement in recognising ambiguous Mooney images after brief exposure to the source image, suggesting intact priors among autistic individuals (Cruys et al., 2018), and similar MMN responses to auditory rhythmic deviants, which suggests typical habituation and prediction (Knight et al., 2020).

Though most of the Bayesian and predictive coding models of autism were initially used to describe low-level sensory perception among autistic individuals, they may be applicable to high-level cognitive processes in autism such as learning of statistical regularities and associations as well as prediction and decision-making. Some studies reported that relative to neurotypical individuals, autistic individuals showed differential neural responses to statistical learning of a continuous auditory stream (Scott-Van Zeeland et al., 2010a, 2010b; Wagley et al., 2020) and poorer learning of cue-outcome associations that are needed to make accurate predictions across various paradigms (Amoruso et al., 2019; Fogelson et al., 2019; Greene et al., 2019; Lawson et al., 2017; Sapey-Triomphe et al., 2021a, 2021b), pointing to difficulties autistic individuals may have with extracting regularities from the input and having higher precision to prediction errors. Those models have also been used to understand whether learning differs when the environment changes, which is typically assessed using a probabilistic reversal learning task: learners first learn the contingencies of two cues and their outcomes over many trials (e.g., Cue A is associated with a reward at an 80:20 reinforcement schedule) after which the contingencies change (e.g., now Cue B rewards at an 80:20 reinforcement schedule). Previous studies found that autistic individuals showed similar performance as neurotypical individuals during the initial learning stage (Costescu et al., 2015; D’Cruz et al., 2013), but their performance was affected more during the reversal stage than neurotypical individuals (e.g., by making more perseverative errors, i.e., selecting the cue that was previously reinforced) (Crawley et al., 2020; Robic et al., 2015; South et al., 2012). Note, however, like the low-level sensory studies, mixed findings have been reported: relative to neurotypical individuals, autistic individuals have shown similar performance on various statistical learning and implicit learning tasks (Brown et al., 2010; Nemeth et al., 2010; Obeid et al., 2016; Zwart et al., 2018a, 2018b); learning cue-outcome associations that have a reinforcement schedule of at least 70:30 (Retzler et al., 2021; Sapey-Triomphe et al., 2021a, 2021b; Solomon et al., 2011); and during both the initial and reversal stages of probabilistic reversal learning (Manning et al., 2017). Thus, the empirical support for the Bayesian and predictive coding models of autism is mixed and remains to be determined.

The inconsistencies for the support for the Bayesian and predictive coding models of autism may be due in part to participant heterogeneity across the different studies (e.g., age differences, whether the autistic and NT participants were properly matched, whether autistic traits or autism diagnosis was used as comparison, etc.). Moreover, methodological differences such as the use of different tasks and how performance on the tasks were measured (i.e., using behavioural accuracy, reaction time, computational modelling, or neuroimaging) across the different studies render it difficult to compare and pinpoint exactly whether support for the models is warranted. It is beyond the scope of this manuscript to examine the inconsistencies empirically. Instead, we tested support for the models by examining an aspect often neglected in previous studies.

A common limitation across most of the statistical learning and cue-outcome association learning tasks above is that the target cue to be tracked is often straightforward (e.g., in a cue-outcome association task, the cues may be two auditory tones of different frequencies; in a probabilistic reversal learning task, learners learn the reward outcome of two different coloured boxes). This is not representative of the real-life cue-outcome associations one must learn to guide our predictions, which are often complex given the many-to-one relationship between cues and outcomes. For example, when trying to predict if it will rain, in addition to looking at the colour of the clouds, one may smell the air for a ‘metallic’ smell; see whether the shapes of pinecones are closed; and determine whether cows around them are lying down according to a folklore. But not all the cues are equally predictive (or indeed valid), and so an ideal learner will need to infer over time which cues are more reliable than others in their association with the outcome. While this has not been examined directly, there are reasons to suspect that autistic individuals may find this cue-inferencing process to be more challenging than neurotypical individuals. Some researchers have noted that autistic individuals tend to associate only one perceptual cue of a complex cue stimulus with a response, a phenomenon termed ‘stimulus overselectivity’ (Lovaas et al., 1979; Ploog, 2010), which may hinder their ability to infer the correct cue(s) to learn in a predictive context. Moreover, autistic individuals’ tendency to commit perseverative errors in probabilistic reversal learning tasks suggests that they may find it difficult to switch between different cues to determine the most predictive one.

The present experiments directly addressed the limitation of straightforward cue-outcome relationships seen in previous studies by examining whether one’s level of autistic traits will influence their ability to infer and learn the target cue from a stimulus with multi-faceted cues and its associations with the outcome in a probabilistic learning task. Specifically, the stimuli were auditory pseudospeech of various length and acoustic manipulation and the target cue was associated with the outcome deterministically (i.e., the cue is 100% predictive of the outcome) or probabilistically (i.e., the cue is 75% predictive of the outcome). While the present stimuli are still relatively simple compared to those encountered in real life, they are arguably more complex than those seen in previous studies where the cues in the stimuli differed in one dimension only (e.g., tone frequency, colour, etc.). Participants were either told explicitly to determine the cue-outcome relationships in Experiment 1 (though note that they were not specifically told which cue to focus on) or they were exposed to the cue-outcome relationship implicitly via a cover task in Experiment 2. The instruction manipulation was motivated by recent suggestions that atypical prediction processing may be more likely observed among autistic individuals when the cue-outcome associations are low in salience (Amoruso et al., 2019; Cannon et al., 2021; Westerfield et al., 2015). Across both experiments, if individuals with high autistic traits have difficulty inferring cues and learning cue-outcome relationships, then there should be a negative relationship between autistic traits and accuracy on the probabilistic learning task.

## Experiment 1 (Explicit)

### Methods

#### Participants

A total of 101 individuals (Female *n* = 54, Male *n* = 44, Other/Non-binary *n* = 3) participated in the study, about a third of whom completed the experiment in-person in the lab (*n* = 29) and the rest completed the experiment online (*n* = 72)^1^. All of them were adults; their age ranged between 16 and 58 years (M = 34.71, SD = 12.26). About a fifth of the participants reported to have a clinical diagnosis of autism spectrum conditions (ASC) (lab *n* = 17; online *n* = 3) but due to the anonymity of the online experiment, we could only confirm the diagnosis of the lab participants by verifying their clinical diagnostic report. Three online participants reported that they ‘Don’t Know’ if they have a clinical diagnosis of ASC, whereas the rest of the lab (*n* = 12) and online (*n* = 67) participants reported they do not. Regardless of their diagnosis status, we used the Autism-Spectrum Quotient (AQ) (Baron-Cohen et al., 2001) to measure their autistic traits: the AQ scores for our sample ranged between 2 and 47 (M = 24.65, SD = 11.02). We recruited participants for the lab study via our participant database, flyers/posters, and social media while online participants were recruited using Prolific. An additional seven participants completed the online study but were excluded from the analysis as they did not meet the attention check threshold (see Tasks subsection below). The study protocol was reviewed and approved by the University Research Ethics Committee (UREC) at the University of Reading. All participants provided their written informed consent prior to their participation.

#### Tasks

Data collection for the lab study was conducted using PsychoPy (Peirce, 2007) whereas online data collection was done using Gorilla (Anwyl-Irvine et al., 2020). All participants completed two tasks: (i) probabilistic learning task, and (ii) perception task.

##### Probabilistic Learning Task

There were two phases in the probabilistic learning task: learning phase and test phase. At the start of the learning phase, participants were told that they would be presented with someone practising a magic trick of reciting a spell and pulling an object out from a top hat and that she might get it wrong sometimes. Participants were told to decide which of four objects displayed would be chosen after each spell within 1.5 s, after which regardless of whether participants responded, they would be shown the object that had been pulled out (i.e., they would be given feedback). Participants were instructed to try and figure out how the magic trick worked, and to guess their responses if they were unsure. The learning phase was divided into two blocks (first half vs. second half) to examine learning over time. The test phase that followed had the same format as the learning phase except there was only one test block, no time limit to respond, and no feedback.

The spells were presented auditorily and consisted of strings of 3–7 nonsense syllables (e.g., ‘mot pel pel jig’, ‘dag ruk jig jig mot pel’, etc.) synthesised using Mac OS X Speech Service with a female voice. We levelled the pitch contour of each spell and then further manipulated the spells in one of four ways such that as the spell unfolds, the pitch contour rises (pitch-rise), falls (pitch-fall), or the tempo of the spell increases (tempo-fast) or decreases (tempo-slow). Pitch manipulation was done on Praat (Boersma & Weenink, 2013), which involved changing the shape of the rise or fall by approximately two semitones, whereas tempo manipulation was performed on Audacity (Audacity Team, 2018), which involved increasing/decreasing the tempo of the second half of the spell by 40% using the built-in function in Audacity. Each of the spell manipulation (pitch-rise, pitch-fall, tempo-fast, and tempo-slow) was associated with one of four objects, and we created two languages—Language A and Language B—that differed in the spell manipulation-object assignment. Participants were randomly assigned to one language at the start of the experiment. Within each language, two of the spell manipulation-object associations—one pitch- and one tempo-manipulation—were deterministic (i.e., the spell manipulation was 100% predictive of the object) whereas the other two were probabilistic (i.e., the spell manipulation was predictive of the object 75% of the time). In total, 32 unique spells (4 spell manipulations × 8 spells) were presented twice in the learning phase whereas in the test phase, 20 unique spells (4 spell manipulations × 5 spells), different from that encountered during the learning phase, were presented twice. Importantly, at no point during this task were participants told what cues to attend to or how the spell manipulation-object associations were defined.

Participants completed four practice trials in the learning phase and two practice test trials in the test phase prior to the main task to ensure they understood the instructions. To ensure attentiveness and to exclude bot responses, we included catch trials in the online study: participants were instructed to press a particular key when they heard spells produced by a male voice. Participants who scored less than 70% correct on the catch trials were excluded from data analysis.

##### Perception Task

Following the probabilistic learning task, participants completed a perception task, which assessed their ability to discriminate the pitch and tempo manipulations, and thus acted as a control task to exclude the possible confounding effects of perceptual ability on the probabilistic learning task. A same/different paradigm was used: participants were presented with pairs of disyllabic stimuli of different syllables within each pair (e.g., “mot-pel” vs “jig-mot”) with an inter-stimulus-interval of 500 ms. They were instructed to determine whether the pairs were identical in their acoustic cues and were explicitly told to not base their judgment on the syllables themselves. For the same trials, pairs of ‘base’ stimuli (i.e., flat pitch, no tempo manipulation) were presented. For the different trials, ‘base’ stimuli were paired with one of the four spell manipulations (pitch-rise, pitch-fall, tempo-rise, and tempo-fall). Participants completed 32 trials in total—16 same trials, 16 different trials (4 trials for each spell manipulation)—presented in a random order.

#### Procedure

Participants first completed a questionnaire on their demographic information and then the AQ questionnaire. Then, participants completed the probabilistic learning task followed by the perception task. The entire study took approximately 40 min to complete, and participants received monetary compensation for their time.

#### Data Analysis

Prior to conducting a formal data analysis, visual inspection on the probabilistic learning task performance revealed that some of the participants performed poorly, suggesting floor effects. Similar to previous studies that removed participants who failed to show learning in probabilistic tasks (Solomon et al., 2011), we removed participants who scored at or below 0.25 proportion correct (i.e., chance level for a 4-alternative forced choice task) in the test phase (*n* = 15). Thus, the data analysis for Experiment 1 reported below is based on a final sample of 86 participants. Analysis on the entire sample (*n* = 101) can be found in Supplementary Section S1, and generally the same pattern of findings was found.

##### Perception Task

For each participant, d-prime (d’) scores for the pitch and tempo manipulations were calculated separately. Extreme values of 0 and 1 for Hit and False Alarm rates were adjusted upwards and downwards by 0.01, respectively (Macmillan & Creelman, 2005). The d’ scores were compared against zero to determine whether participants reliably discriminated the pitch and tempo manipulations. We also compared the pitch d’ and tempo d’ against each other using a paired *t*-test to determine if there were differences in discrimination ability between the two. Each d’ was correlated with AQ to examine if discrimination ability differed as a function of autistic traits.

##### Probabilistic Learning Task

To model participants’ responses in the learning phase, we fitted a binomial mixed effects model to the data, with the dependent variable being a binary variable (Correct/Incorrect, with Correct being the object that is most likely associated with the spell manipulation). As fixed effects, we entered Pitch d’, Tempo d’, AQ, Block (Block 1 vs. Block 2), Type (Deterministic vs. Probabilistic) and all the possible interactions between AQ, Block and Type. We included Language as a fixed effect initially but dropped it from the final model as it did not significantly affect the results. As random effects, we entered by-subject and by-item random intercepts and by-subject random slope for Block and Type.

Participants’ responses in the test phase were also modelled using a binomial mixed effects model, with the following fixed effects: Pitch d’, Tempo d’, AQ, Type (Deterministic vs. Probabilistic) and AQ × Type. Language was initially included as a fixed effect but was removed as it did not significantly affect the results. We included subject- and item-level random intercepts, and by-subject random slopes for Type.

In the models above and subsequent models reported in this paper, all continuous variables were mean centred, and all categorical variables were effect-coded. As a measure for effect size, we reported odds ratio, in which magnitude further away from 1.0 (either greater or less) is interpreted as a stronger association. The models were fitted using the *lme4* package (Bates et al., 2015) and the statistical significance of each fixed effect was determined using the function *Anova()* from the *car* package (Fox & Weisberg, 2019). Pairwise comparisons were conducted using the *emmeans* package (Lenth, 2019). All the predictors in each model had a low variance inflation factor (VIF) value (< 3) as assessed using the function *check_collinearity()* from the *performance* package (Lüdecke et al., 2021), suggesting no issue with multicollinearity in each model.

## Results and Discussion

Participants as a group reliably discriminated the pitch and tempo manipulations (pitch d’: M = 1.90, SD = 1.06, *t*(85) = 16.62, *p* < 0.001; tempo d’: M = 2.60, SD = 1.15, *t*(85) = 20.95, *p* < 0.001), and their discrimination ability was better in the tempo trials than in the pitch trials [*t*(85) = 5.57, *p* < 0.001]. Higher AQ scores were significantly associated with lower pitch d’, that is, lower pitch discrimination ability [*r*(84) = − 0.25, *p* = 0.019] but AQ scores were not associated with tempo d’ [*r*(84) = − 0.17, *p* = 0.125].

**Fig. 1:**
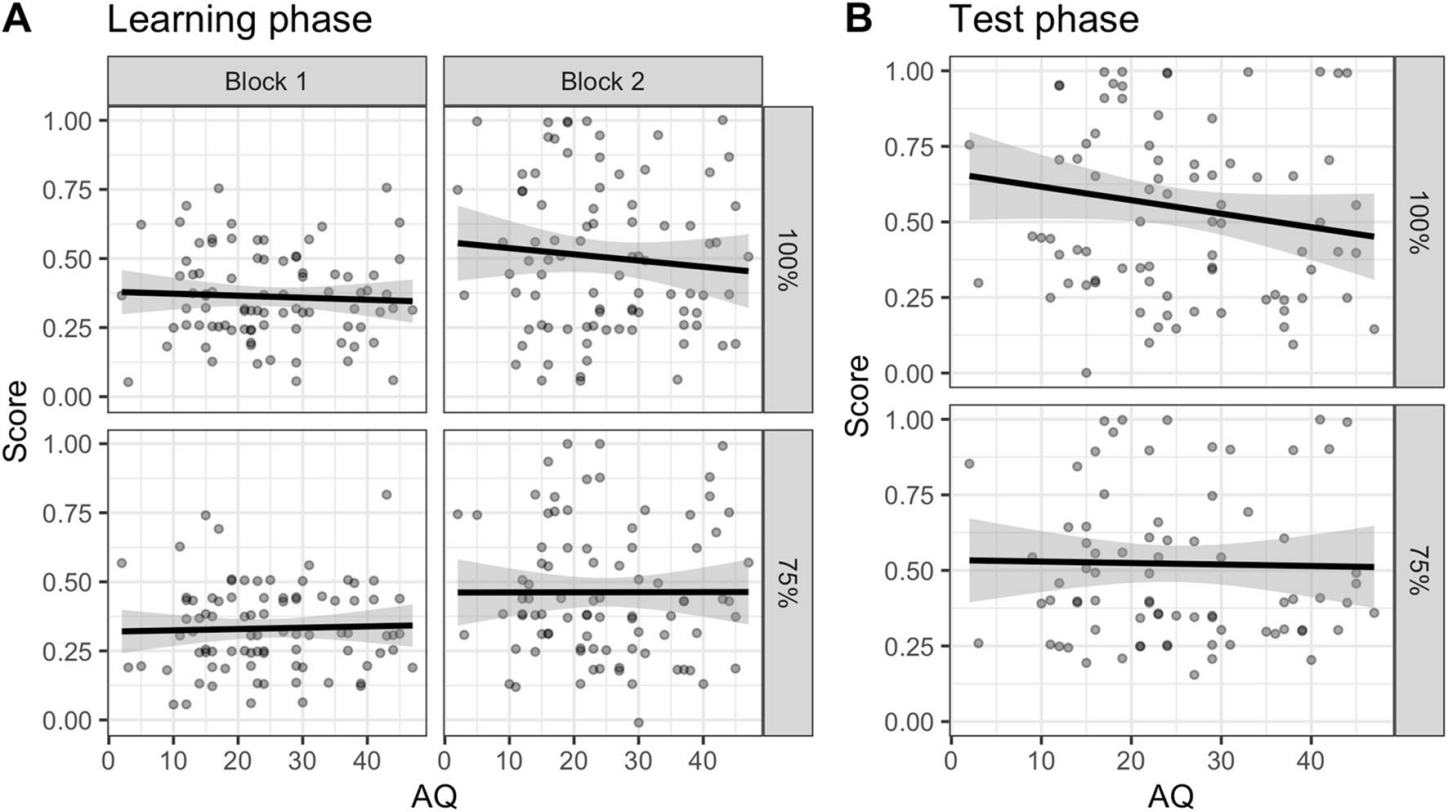
Proportion correct on the deterministic (100%) and probabilistic (75%) trials as a function of autistic traits (AQ) for the learning phase by block **A** and test phase **B** after removing participants who showed floor effects

In the learning phase model (see Table 1 for the model output), Block was a significant predictor [χ^2^(1) = 32.08, *p* < 0.001], with accuracy on the second half greater than the first (*z* = 5.66, *p* < 0.001), suggesting learning over time. Type was also significant [χ^2^(1) = 4.92, *p* = 0.027], suggesting that performance on the Deterministic trials were more accurate than the Probabilistic trials (*z* = 2.21, *p* = 0.027). Importantly, none of the predictors involving AQ were significant (AQ: χ^2^(1) = 0.16, *p* = 0.685; AQ × Block: χ^2^(1) = 0.22, *p* = 0.638; AQ × Type: χ^2^(1) = 1.33, *p* = 0.249; AQ × Block × Type: χ^2^(1) = 0.39, *p* = 0.533), suggesting that there was no statistically significant effect of autistic traits on learning the two types of associations (see Fig. 1A).

**Table 1:** Model output for the learning phase of Experiment 1 (Explicit)

Predictor	Estimate	SE	*z*	*p*	Odds ratio
Intercept	− 0.36	0.10	− 3.75	< .001	0.70
Pitch d’	− 0.05	0.08	− 0.64	.521	0.95
Tempo d’	0.03	0.07	0.48	.633	1.03
AQ	0.00	0.01	− 0.41	.685	1.00
Block	− 0.65	0.12	− 5.66	< .001	0.52
Type	0.18	0.08	2.22	.027	1.20
AQ × Block	0.00	0.01	0.47	.638	1.00
AQ × Type	− 0.01	0.01	− 1.15	.249	0.99
Block × Type	− 0.10	0.12	− 0.80	.427	0.91
AQ × Block × Type	0.01	0.01	0.62	.533	1.01

Final model: Correct ~ Pitch d’ + Tempo d’ + AQ*Block*Type + (1 + Block + Type|Participant) + (1|Item)

In the test phase model (see Table 2 for the model output), just as in the learning phase, neither AQ [χ^2^(1) = 0.02, *p* = 0.877] or AQ × Type [χ^2^(1) = 2.90, *p* = 0.089] were significant, suggesting that the accuracy of deterministic and probabilistic trials was not related to autistic traits (see Fig. 1B).

**Table 2:** Model output for the test phase of Experiment 1 (Explicit)

Predictor	Estimate	SE	*z*	*p*	Odds ratio
Intercept	0.38	0.18	2.12	.034	1.47
Pitch d’	0.34	0.19	1.79	.073	1.40
Tempo d’	0.11	0.17	0.63	.530	1.11
AQ	0.00	0.02	− 0.17	.867	1.00
Type	0.22	0.16	1.40	.161	1.25
AQ × Type	− 0.02	0.01	− 1.70	.089	0.98

Final model: Correct ~ Pitch d’ + Tempo d’ + AQ*Type + (1 + Type|Participant) + (1|Item)

Overall, the findings from Experiment 1 revealed that participants, regardless of their levels of autistic traits, had similar performance on the deterministic and probabilistic trials. This appears contrary to the Bayesian and predictive coding accounts of autism (Brock, 2012; Cruys et al., 2014; Lawson et al., 2014; Pellicano & Burr, 2012; Sinha et al., 2014), which would predict that autistic individuals or individuals with higher levels of autistic traits should perform worse on the probabilistic trials than neurotypical individuals. One possibility for this may be our task instructions, that is, participants were explicitly told to figure out the magic trick. Indeed, some have suggested that differential performance in prediction and learning associations among autistic and neurotypical individuals may be more readily observed under more implicit conditions (Cannon et al., 2021). For example, when the association between cue and outcome was made less apparent (e.g., presented in the background), the autistic group were less likely to learn the association (Amoruso et al., 2019). Thus, in Experiment 2, we repeated Experiment 1 with one crucial difference: participants were not told to figure out the magic trick, but instead, they completed a cover task during the learning phase that nonetheless exposed them to the spell manipulation-object associations.

## Experiment 2 (Implicit)

### Methods

#### Participants

Participants consisted of 73 adults (Female *n* = 52, Male *n* = 17, Other/Non-binary *n* = 4), with their ages ranging between 18 and 59 (M = 28.16, SD = 10.19). All participants, none of whom had participated in Experiment 1, were recruited via Prolific and completed the experiment online. About 37% of the participants reported having a clinical diagnosis of ASC (*n* = 27), though this was not verified due to the data collection method. Three participants responded that they ‘Don’t Know’ if they have a clinical diagnosis of ASC, whereas the rest (*n* = 43) reported they do not. Their autistic traits, as measured using the AQ, ranged between 6 and 46 (M = 26.93, SD = 11.84). The study protocol was reviewed and approved by the University Research Ethics Committee (UREC) at the University of Reading. All participants provided their written informed consent prior to their participation.

#### Tasks

Similar to Experiment 1, data collection was conducted using Gorilla and all participants completed two tasks: (i) probabilistic learning task, and (ii) perception task.

##### Probabilistic Learning Task

Participants completed a similar probabilistic learning task as Experiment 1 with one crucial difference during the learning phase: participants were told that the study was on how quickly one processes information and thus, instead of predicting the outcome after each spell, participants completed a cover task during which they had to count the number of syllables in each spell and modify the calculation based on the object that was shown within 5 s. For example, if they heard the spell ‘mot pel jig’ and the leftmost object in the row of four objects were shown, then the correct answer is ‘4’ (3 syllables + 1st object). Thus, participants were not explicitly told to figure out the associations between spells and outcome like in Experiment 1, but they were nonetheless exposed to the associations. The cover task was preceded by four practice trials to ensure they understood the instructions. To ensure attentiveness, catch trials were implemented: participants were told to respond ‘0’ when they heard male spoken spells.

##### Perception Task

The same perception task as in Experiment 1 was used.

#### Procedure

Just like in Experiment 1, participants completed the experiment in the following order: (i) demographic questionnaire; (ii) AQ questionnaire; (iii) probabilistic learning task; and (iv) perception task. The entire study took approximately 40 min to complete, and participants received monetary compensation for their time.

#### Data Analysis

Similar to Experiment 1, we removed participants who scored at or below 0.25 proportion correct (i.e., chance level for a 4-alternative forced choice task) in the test phase (*n* = 28), leaving the data analysis for Experiment 2 below to be based on 45 participants. Analysis on the entire sample (*n* = 73) is reported in Supplementary Sections S2 and S3, and we found similar pattern of findings.

##### Perception Task

We analysed participants’ perception task scores in the same manner as in Experiment 1, that is, pitch d’ and tempo d’ were calculated for each participant and we assessed whether the two (i) were above zero; (ii) different from each other; and (iii) correlated with AQ.

##### Probabilistic Learning Task

We used a binomial mixed effects model to model their accuracy on the cover task, with the dependent variable being a binary variable (Correct/Incorrect), and we entered Pitch d’, Tempo d’, AQ, Block (Block 1 vs. Block 2) and AQ × Block as fixed effects (Type was not included here as it was irrelevant to the cover task). We also included Language (A vs. B) initially, but it was dropped as it did not significantly affect the results. We included by-subject and by-item intercepts and by-subject random slope for Block as random effects.

The same analysis as Experiment 1 for the test phase was used to model the test phase data of Experiment 2: we entered Pitch d’, Tempo d’, AQ, Type (Deterministic vs. Probabilistic) and AQ × Type as fixed effects, and subject- and item-level random intercepts and by-subject random slopes for Type as random effects. We also compared the test phases across experiments^2^ using the following model: as fixed effects, we entered Pitch d’, Tempo d’, AQ, Type (Deterministic vs. Probabilistic), Experiment (Explicit vs. Implicit) and all possible interactions between the latter three, and as random effects, we included by-subject and by-item intercepts as well as by-subject random slopes for Type. Language was not included as a fixed effect in the final model as it did not significantly affect the results.

## Results and Discussion

Similar to Experiment 1, participants discriminated the pitch and tempo manipulations above chance (pitch d’: M = 2.01, SD = 0.73, *t*(44) = 18.54, *p* < 0.001; tempo d’: M = 2.25, SD = 1.42, *t*(44) = 10.58, *p* < 0.001). However, unlike Experiment 1, their performance between the two was similar [*t*(44) = 0.94, *p* = 0.352], and their AQ scores were not significantly associated with their discrimination ability (pitch: *r*(43) = 0.18, *p* = 0.240; tempo: *r*(43) = − 0.25, *p* = 0.101).

**Fig. 2:**
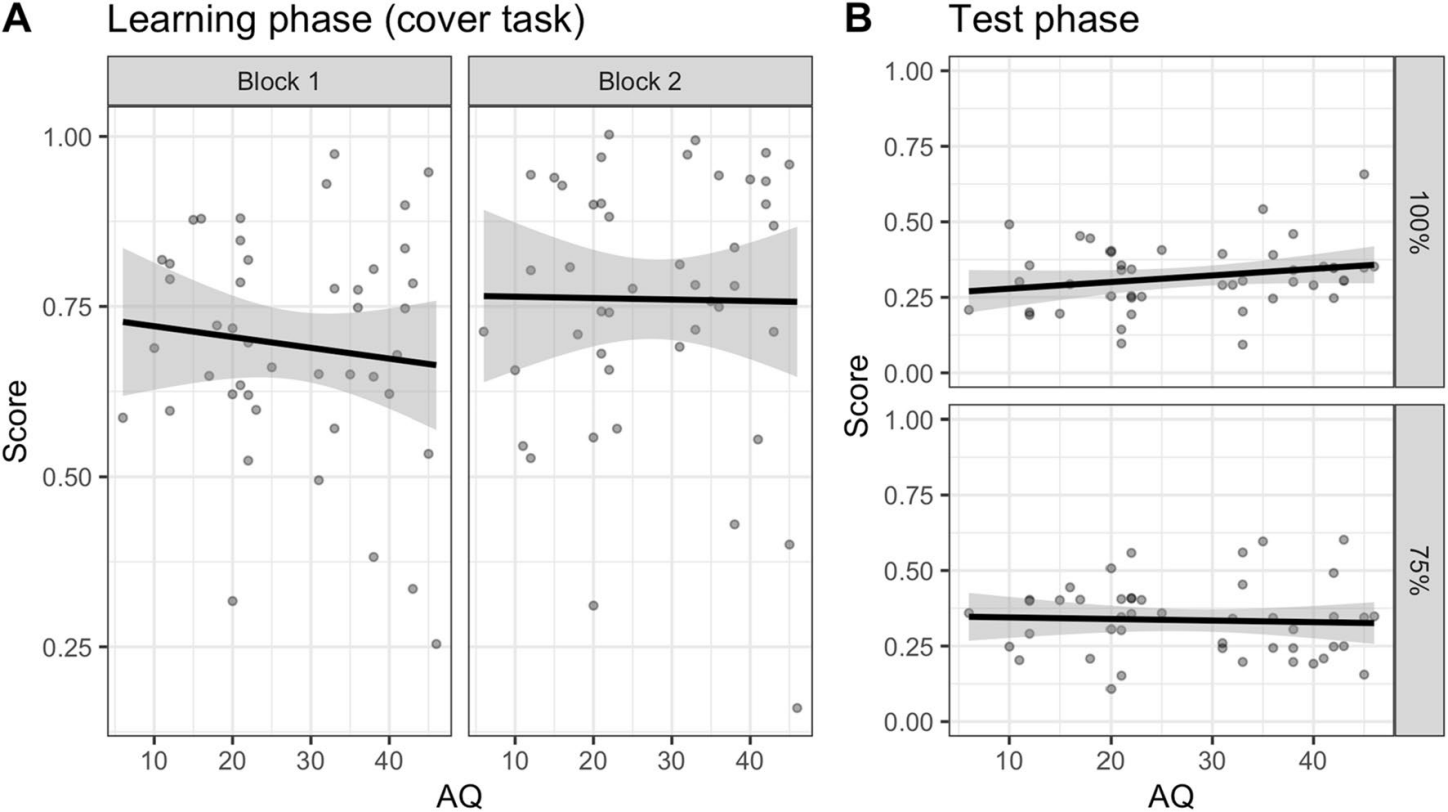
Proportion correct on the cover task by block **A** and on the deterministic (100%) and probabilistic (75%) trials in the test phase **B** as a function of autistic traits (AQ) after removing participants who showed floor effects

Focusing just on their performance on the cover task during the learning phase (see Table 3 for the model output), Pitch d’ was a significant predictor [χ^2^(1) = 8.98, *p* = 0.003]: higher pitch perception ability was associated with greater accuracy on the cover task (*B* = 0.57, SE = 0.19, *z* = 3.00, *p* = 0.003). Block was also a significant predictor [χ^2^(1) = 17.41, *p* < 0.001], with accuracy on the second half greater than the first (*z* = 3.92, *p* < 0.001). There was no significant effect of AQ on the accuracy of the cover task either overall [χ^2^(1) = 0.74, *p* = 0.389] or by Block [χ^2^(1) = 0.96, *p* = 0.327], suggesting that participants, regardless of their levels of autistic traits, showed practice-related improvement (see Fig. 2A).

**Table 3:** Model output for the learning phase (cover task) of Experiment 2 (Implicit)

Predictor	Estimate	SE	*z*	*p*	Odds ratio
Intercept	1.32	0.23	5.66	< .001	3.75
Pitch d’	0.57	0.19	3.00	.003	1.76
Tempo d’	− 0.17	0.10	− 1.69	.092	0.84
AQ	− 0.01	0.01	− 0.86	.389	0.99
Block	− 0.59	0.14	− 4.17	< .001	0.55
AQ × Block	− 0.01	0.01	− 0.98	.327	0.99

Final model: Correct ~ Pitch d’ + Tempo d’ + AQ*Block + (1 + Block|Participant) + (1|Item)

The model for the test phase (see Table 4) revealed that, similar to Experiment 1, there was also no significant effect of AQ [χ^2^(1) = 0.66, *p* = 0.416] or AQ × Type [χ^2^(1) = 1.28, *p* = 0.259], suggesting that autistic traits did not affect performance on the deterministic and probabilistic trials (see Fig. 2B).

**Table 4:** Model output for the test phase of Experiment 2 (Implicit)

Predictor	Estimate	SE	*z*	*p*	Odds ratio
Intercept	− 0.75	0.06	− 12.94	< .001	0.47
Pitch d’	0.08	0.07	1.18	.237	1.09
Tempo d’	0.03	0.04	0.83	.405	1.03
AQ	0.00	0.00	0.82	.411	1.00
Type	− 0.10	0.13	− 0.78	.436	0.91
AQ × Type	0.01	0.01	1.14	.254	1.01

Final model: Correct ~ Pitch d’ + Tempo d’ + AQ*Type + (1 + Type|Participant) + (1|Item)

**Fig. 3:**
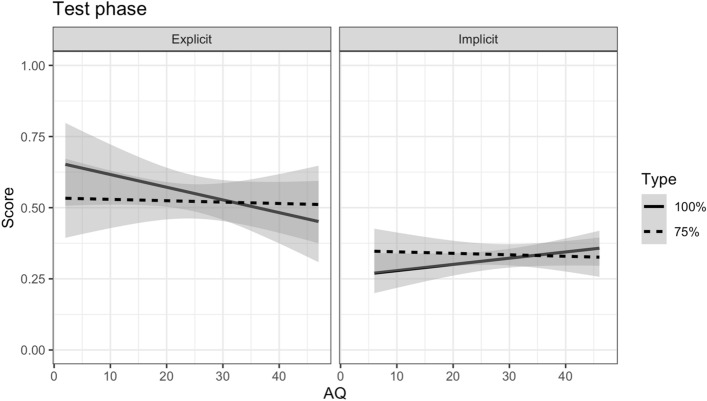
Fitted data of proportion correct on the deterministic (100%, solid line) and probabilistic (75%, dashed line) trials as a function of autistic traits (AQ) by Experiment (Explicit = Experiment 1; Implicit = Experiment 2) after removing participants who showed floor effects

When we compared the two test phases across the experiments (see Table 5), we found that Pitch d’ was a significant predictor [χ^2^(1) = 5.57, *p* = 0.018] such that higher pitch perception ability was associated with greater performance (*B* = 0.28, SE = 0.12, *z* = 2.36, *p* = 0.018). We speculate that the pitch d’ score may reflect reasoning skills, given that previous studies have found an association between pitch perception and nonverbal reasoning abilities (Chowdhury et al., 2017), which is arguably important in a task like the present study. Though note, however, that pitch perception cannot bias the learning of different types of association in this study as both deterministic and probabilistic associations had one pitch manipulation each. There was also a significant effect of Experiment [χ^2^(1) = 25.86, *p* < 0.001], with overall performance on the explicit experiment higher than the implicit experiment (*z* = 5.08, *p* < 0.001). While there was no effect of AQ [χ^2^(1) = 0.00, *p* = 0.945], AQ × Type [χ^2^(1) = 0.22, *p* = 0.642], or AQ × Experiment [χ^2^(1) = 0.16, *p* = 0.687], the three-way interaction between AQ, Type and Experiment was marginally significant [χ^2^(1) = 3.61, *p* = 0.058, see Fig. 3]. Subsequent comparisons revealed a marginal difference in the effect of AQ across Type, with the estimated AQ slope for the deterministic trials slightly more negative than that for the probabilistic trials for the explicit experiment (*z* = 1.91, *p* = 0.057) but not the implicit experiment (*z* = 0.92, *p* = 0.360). The difference in the effect of AQ across experiments did not differ by type (Deterministic: *z* = 1.14, *p* = 0.256; Probabilistic: *z* = 0.49, *p* = 0.625).

**Table 5:** Model output for comparison between test phases Experiment 1 (Explicit) and Experiment 2 (Implicit)

Predictor	Estimate	SE	*z*	*p*	Odds ratio
Intercept	− 0.24	0.12	− 2.10	.036	0.78
Pitch d’	0.28	0.12	2.36	.018	1.32
Tempo d’	0.07	0.09	0.83	.407	1.08
AQ	0.00	0.01	− 0.07	.945	1.00
Type	0.05	0.11	0.43	.664	1.05
Experiment	1.12	0.22	5.09	< .001	3.08
AQ × Type	0.00	0.01	− 0.46	.642	1.00
AQ × Experiment	− 0.01	0.02	− 0.40	.687	1.00
Type × Experiment	0.29	0.22	1.30	.193	1.33
AQ × Type × Experiment	− 0.04	0.02	− 1.90	.058	0.96

Final model: Correct ~ Pitch d’ + Tempo d’ + AQ*Type*Experiment + (1 + Type|Participant) + (1|Item)

## General Discussion

Making correct inferences and predictions is vital and permeates all aspects of our lives. To make an optimal prediction, one needs to keep track of the statistical regularities in the environment including cue-outcome associations. It is unclear from previous studies whether autistic individuals may have more difficulties learning statistical regularities in the environment (Brown et al., 2010; Nemeth et al., 2010; Scott-Van Zeeland et al., 2010a, 2010b; Wagley et al., 2020), though it seems that their ability to do so is likely preserved when the contingencies are relatively high (Sapey-Triomphe et al., 2021a, 2021b; Solomon et al., 2011). However, those previous studies tend to be simplistic in that the target cue to be tracked is relatively straightforward and obvious, which is unlike real-life situations given the many-to-one relationship between cues and outcomes, and so learners need to infer which cues are more reliable than others. We addressed this limitation directly in this study by comparing individuals with varying levels of autistic traits on learning cue-outcome associations that are either deterministic or probabilistic from a set of many possible cues. Participants were either explicitly told to determine the cue-outcome associations (Experiment 1) or performed a cover task that exposed them to the associations implicitly (Experiment 2).

Our findings revealed no significant effect of autistic traits on inferring the appropriate cue from a set of many possible cues to learn deterministic or probabilistic contingencies.^3^ This study thus extends the findings of previous studies that typically used a case–control approach, unlike the present study, which found no group differences among autistic and neurotypical individuals in learning single cue-outcome associations that are at least 70% predictive (Costescu et al., 2015; D’Cruz et al., 2013; Sapey-Triomphe et al., 2021a, 2021b; Solomon et al., 2011). In our study, the effect of autistic traits was not observed in either the explicit or implicit version of the task, which contradicts some suggestions that differences in prediction among autistic and neurotypical individuals are more likely observed when the cue-outcome associations are less apparent or have less predictive salience (Amoruso et al., 2019; Cannon et al., 2021; Westerfield et al., 2015).

Overall, then, our findings contradict the predictions of Bayesian and predictive coding accounts of autism. Regardless of whether autistic individuals have attenuated priors (Pellicano & Burr, 2012), higher sensory precision (Brock, 2012), atypical precision of prediction errors (Cruys et al., 2014; Lawson et al., 2014), or difficulties learning regularities needed to make predictions (Sinha et al., 2014), the different models would predict that autistic traits should affect the learning of probabilistic associations more so than the deterministic associations given the occasional incorrect feedback in the probabilistic trials. This was not what we found; if anything, there was some indication that autistic traits negatively affected the learning of deterministic associations more so than probabilistic associations when learners were explicitly told to figure out the associations. However, the interaction was only marginally significant, and thus should be interpreted with caution. We consider some possibilities for our findings and the limitations of the present study below.

One possibility relates to our approach of using autistic traits within the general population (some of whom self-reported to be autistic albeit most were not) rather than using a case–control design with autistic individuals with a clinical diagnosis vs. neurotypical individuals commonly seen in autism research. This is one of the limitations of the present study as the two approaches are not equivalent; indeed, some researchers have warned against conflating high levels of autistic traits with autism (Lord & Bishop, 2021; Sasson & Bottema-Beutel, 2021). In the literature, previous studies have reported groups differences in case–control studies of various statistical learning and prediction task (Crawley et al., 2020; Robic et al., 2015; Scott-Van Zeeland et al., 2010a, 2010b; Wagley et al., 2020) whereas those that examined autistic traits failed to find any significant effects of autistic traits (Parks et al., 2020; Retzler et al., 2021). However, there are many counter-examples of this: some studies have found no group differences in case–control studies (Barnes et al., 2008; Haebig et al., 2017; Manning et al., 2017; Nemeth et al., 2010; Sapey-Triomphe et al., 2021a, 2021b; Zwart et al., 2018b) and some have found an effect of autistic traits on statistical learning and prediction (Nassar & Troiani, 2021; Parks et al., 2020). Moreover, some studies that have considered both approaches have managed to replicate the same findings across case–control design and autistic traits (Cruys et al., 2018; Lawson et al., 2017; Pell et al., 2016), suggesting that both approaches may yield the same conclusion. Thus, while we cannot definitively rule out that our findings are due to the present study using autistic traits rather than a case–control approach,^4^ we think it is unlikely the main reason for our findings.

Another possibility concerns our task and measurement of statistical learning. The novel task that we have created may have been too difficult, especially the implicit task, leading many to show floor effects. However, comparing the analysis with the full sample and with those that performed above chance level showed similar findings in that autistic traits did not affect participant’s performance on the deterministic and probabilistic associations. Thus, the issue of task difficulty may contribute but is unlikely to be the main cause for our findings. Setting task difficulty aside, the measurement used, that is, behavioural accuracy, may not be sensitive enough to detect the effects of autistic traits. Specifically, such crude behavioural measures do not capture the mechanism or compensatory strategies one may use to base their response, which can be revealed by computational modelling instead. Previous studies have reported group differences in certain model parameters (e.g., the learning rate) in tasks such as the probabilistic reversal learning task (Crawley et al., 2020; Lawson et al., 2017), though others using a similar task have failed to find any group differences or effects of autistic traits on such model parameters (Goris et al., 2021; Manning et al., 2017). Neuroimaging is another sensitive tool that tends to reveal group differences in statistical learning and prediction and help elucidate the underlying atypical mechanism. For example, compared to neurotypical individuals, autistic individuals tend to show less neural activation during statistical learning (Scott-Van Zeeland et al., 2010a, 2010b; Travers et al., 2015) and less differential electrophysiological activity between deviant types of different frequency (Goris et al., 2018). Overall, more studies that incorporate these sensitive measures are needed in the future to clarify the current findings.

Finally, it is possible that perhaps we did not find any effect of autistic traits on statistical learning simply because autism or autistic traits do not influence one’s ability to infer, learn, and predict from statistical regularities. Our findings add to the growing body of research that found similar performances between autistic and neurotypical individuals in various statistical learning and prediction tasks such as probabilistic classification learning (Brown et al., 2010), artificial grammar learning (Brown et al., 2010), auditory segmentation (Haebig et al., 2017), serial reaction time (Barnes et al., 2008; Brown et al., 2010; Nemeth et al., 2010; Zwart et al., 2018a, 2018b), probabilistic reversal learning (Manning et al., 2017), and learning cue-outcome associations (Retzler et al., 2021; Sapey-Triomphe et al., 2021a, 2021b). If this were true, then the current Bayesian and predictive coding theories of autism might need to be refined; perhaps the current accounts may be better suited to explain low-level perceptual and sensory autistic experiences, but not higher-level cognitive aspect of autism. Note, however, that Bayesian theories of autism were used to explain sensory hypersensitivity, which, if true, should predict that higher autistic traits would be associated with better sensory (pitch) perception. This was not the case in our study, which further casts doubt on using such theories to explain autistic experiences.

In conclusion, we did not find any evidence that autistic traits affect the learning of cue-outcome relationships when learners have to infer from a complex set of cues either explicitly or implicitly. While our findings may be due to our methodology (e.g., using autistic traits rather than clinical diagnosis; task difficulty; low sensitivity of our measurement), this study, together with others that similarly found no group differences in statistical learning and prediction across various study designs and tasks, casts doubt on the current Bayesian and predictive coding models of autism. More work is needed to further understand the underlying mechanism of statistical learning and prediction among autistic and neurotypical individuals, which will help refine the current theories of autism.

## Data Availability

The dataset supporting the conclusions of this article is available in https://osf.io/7z5p4/﻿.

## Footnotes

1 The change in data collection format was due to COVID-19 restrictions at the time of conducting this study

2 The learning phases across experiments could not be compared meaningfully as the learning phase in Experiment 2 did not involve participants predicting the outcome after each spell.

3 To confirm our frequentist analyses, we repeated the analyses using Bayesian mixed models (see Supplementary Section S4) and found no evidence for the effect of autistic traits on any of the models, that is, we can be 95% certain that the slope does not differ from 0 given our data.

4 We repeated the analyses using two different case–control approaches: we assigned participants to (i) high vs. low autistic traits group based on the AQ scores (see Supplementary Section S5); and (ii) autistic vs. neurotypical group based on their self-reported autism diagnosis (see Supplementary Section S6). Similar findings as the continuous autistic traits analyses were found, with the exception that in the test phase of Experiment 1 (Explicit), individuals with low autistic traits and neurotypical participants showed better performance in the Deterministic compared to Probabilistic trials whereas individuals with high autistic traits and autistic participants did not. The same pattern was additionally found in the learning phase of Experiment 1, but only in the autism diagnosis analysis. However, these findings should be interpreted with caution given the unequal number of participants across groups in Experiment 1 (Autistic traits group analysis: High autistic group *n* = 22 vs. Low autistic group *n* = 64; Autism diagnosis analysis: Autistic group *n* = 16 vs. Neurotypical group *n* = 70).
